# FIN56, a novel ferroptosis inducer, triggers lysosomal membrane permeabilization in a TFEB-dependent manner in glioblastoma

**DOI:** 10.7150/jca.58500

**Published:** 2021-09-13

**Authors:** Xin Zhang, Yulian Guo, Hao Li, Lizhang Han

**Affiliations:** 1 Department of Neurosurgery, Qilu Hospital of Shandong University and Institute of Brain and Brain-Inspired Science, Shandong University, Jinan, China.; 2 Shandong Key Laboratory of Brain Function Remodeling, Jinan, China.; 3 Department of Neurosurgery, Heze third people's hospital, Heze, China.

**Keywords:** FIN56, Ferroptosis, Lysosomal membrane permeabilization, Glioblastoma

## Abstract

**Objective:** To explore the anti-tumor effect of FIN56, a novel ferroptosis inducer, on glioblastoma and its underlying mechanisms.

**Methods:** Two human glioblastoma cell lines, LN229 and U118 were applied in this study. Anti-tumor effect was measured by CCK-8 assay, EdU assay and cell cycle analysis. Fluorescent probes, immunofluorescence, plasmid transfection, shRNA knocking out, reverse transcription PCR, western blot analysis, and transmission electron microscopy were used to study the underlying mechanisms. At last, a subcutaneous nude mice model was used to study the anti-tumor effect of FIN56 *in vivo*. The GraphPad Prism software program was applied for statistical analysis.

**Results:** FIN56 decreased cell viability, inhibited cell proliferation and caused cell cycle arrest on LN229 and U118 cells. Further study showed that FIN56 induced ferroptosis and induced lysosomal membrane permeabilization in a ferroptosis and transfactor EB dependent manner. Animal study demonstrated that FIN56 inhibited glioma growth and caused ferroptosis *in vivo*.

**Conclusion:** FIN56 is a promising anti-tumor compound.

## Introduction

Glioblastoma (GBM) is one of the most malignant primary tumors in the central nervous system 1. Although a comprehensive treatment including surgical resection, chemo- and radio-therapy exists, the prognosis of patients with GBM still remains poor 1, 2. Apoptosis, a programmed cell death, is one of the most important signaling pathways involved in various anti-cancer treatment 3. But tumor cells will develop resistance to apoptosis after repeated treatment 4.

Except for apoptosis, many programmed cell death modes have been discovered, including autophagy, necroptosis, pyroptosis and ferroptosis 5. Ferroptosis, a programmed cell death denominated by Dixon in 2012, is reported to be involved in various medical diseases, including neurodegeneration, organ failure, and cancer inhibition 6. Ferroptosis is characterized by intracellular iron accumulation and subsequent lipid peroxidation 6, 7. Morphologically, shrunken mitochondria and increased density of the mitochondrial membrane can be detected in cells with ferroptosis 5. Cystine-glutamate antiporter (xCT), glutathione peroxidase 4 (GPX4), ferroptosis suppressor protein 1 (FSP1), and GTP cyclohydrolase-1 (GCH1) are reported to be involved in the regulation of ferroptosis by limiting the production of lipid peroxides 8.

Lysosomal membrane permeabilization (LMP), another programmed cell death mode, is a lysosome dependent cell death process 9. In LMP, impaired lysosomal membrane induces the release of specific lysosomal enzymes into the cytoplasm, which triggers a cascade of regulated cell death signaling 10. Among all the lysosomal enzymes involved in LMP, cathepsin B and cathepsin D are the most important 11. Recently, lysosomes are reported to be involved in the regulation of ferroptosis 12. Gao et al. showed that erastin, a ferroptosis inhibitor, induced LMP, which contributed to ferroptosis 13. Zhou et al reviewed that Stat3-dependent LMP facilitates the induction of ferroptosis 14. But until now, few studies illustrated the relationship between ferroptosis and LMP. The underlying mechanisms are still unknown.

FIN56, a novel ferroptosis inducer, triggers ferroptosis by increasing the degradation of GPX4 15. FIN56 also activates squalene synthase, an enzyme involved in the cholesterol synthesis 16. But whether FIN56 has anti-tumor effect is still unknown. In our study, we tried to study the potential role of FIN56 on GBM. Our results showed that FIN56 inhibited GBM cell growth both *in vitro* and *in vivo*. Besides, FIN56 increased lipid peroxidation and ROS production, which suggested that FIN56 induced ferroptosis in GBM cells. We also found that FIN56 triggered LMP. Further studies demonstrated that FIN56 induced LMP in a transfactor EB (TFEB)-dependent manner. As a result, FIN56 is a potential compound in treating GBM patients.

## Materials and methods

### Cell culture

LN229 and U118, two GBM cell lines, were obtained from the Culture Collection of the Chinese Academy of Sciences (Shanghai, China) and cultured in Dulbecco's modified Eagle's medium (DMEM, ThermoFisher Scientific; Waltham, MA, USA) supplemented with 10% fetal bovine serum (FBS; ThermoFisher Scientific).

### Cell viability assay

GBM cell lines LN229 and U118 cells were plated into 96-well plates and incubated overnight. Different doses of FIN56 (0, 0.1, 0.5, 1.0, 2.0 4.0 and 8.0 µM) was added to wells. 24 h later, CCK-8 solution (Dojindo, Kumamoto, Japan) was added to each well. 2 h later, samples were measured at 450 nm on a microplate reader (PerkinElmer; San Jose, CA, USA).

### EdU assay

An EdU Assay Kit (Ribobio; Guangzhou, China) was used to test cell proliferation after different treatment. LN229 and U118 cells were seeded into 24-well, flat-bottomed plates and incubated overnight. Then cells were treated with 1 μM FIN56 or DMSO. 24 h later, cells were incubated with EdU according to the manufacturer's instructions. EdU positive cells were calculated under fluorescence microscopy (Leica DMi8; Wetzlar, Germany).

### Cell cycle analysis

LN229 and U118 cells were seeded into 6-well, flat-bottomed plates and incubated overnight. After treatment with 1 μM FIN56 or DMSO for 24 h, LN229 and U118 cells were collected and fixed with 70% ethanol. Then cells were incubated with propidium iodide (PI) supplemented with RNase (Becton Dickinson, San Diego, CA). Cell cycle analysis was performed on a C6 flow cytometer (BD Biosciences; San Jose, CA, USA).

### Lipid peroxidation and ROS assay

LN229 and U118 cells were seeded into 6-well, flat-bottomed plates and incubated overnight. After treatment with 1 μM FIN56 or DMSO for 24 h, intracellular levels of lipid peroxidation/ROS were assessed using BODIPY581/591 C11/CellRox green dye (ThermoFisher Scientific). BODIPY581/591 C11 or CellRox green dye (5 μM; diluted in DMEM with 10% fetal bovine serum) was added to LN229 and U118 cells. Then images were obtained under confocal microscopy (Leica SP5 Confocal Microscope, Leica).

### Immunofluorescence

LN229 and U118 cells were treated with 1 μM FIN56 or DMSO for 24 h. Then cells were fixed with 4% paraformaldehyde, permeabilized with 0.3% Triton X-100, and incubated with 4-Hydroxynonenal (4-HNE, a lipid peroxidation marker) primary antibody (Abcam; Cambridge, UK) and Alexa Fluor 594 conjugated goat anti-rabbit secondary antibody (Abcam). Images were taken under a Leica TCS SP5 Confocal Laser Scanning Microscope (Leica Microsystem).

### Transmission electron microscopy

The ultrastructure of glioma cells was observed under transmission electron microscopy after LN229 and U118 cells were treated with 1 μM FIN56 or DMSO. Experiments were carried out as described previously 2. Images were obtained using a JEM-1200EX II electron microscope (JEOL; Tokyo, Japan).

### Lysosomal membrane stability

Acridine orange (AO; Sigma-Aldrich, USA) staining and GFP‐fused galectin 3 (Obio, Shanghai, China) transient transfection were used to test lysosomal membrane stability.

After being incubated with AO (5 μg/mL) for 15 min, LN229 and U118 cells were treated with 1 μM FIN56 or DMSO. Then images were obtained by a Leica DMi8.

Lipofectamine 2000 reagent (ThermoFisher Scientific) was used for transient transfections. 48 h later, transfected cells were treated with 1 μM FIN56 or DMSO for 24 h. Images were taken by a Leica SP5 Confocal Microscope.

### Transfection of shRNA

LN229 and U118 cells were transfected with 20 nM shRNA by using lipofectamine 2000 (ThermoFisher Scientific) according to the manufacturer's protocol. Sequences for the shRNAs were the following: negative control, 5'-UUCUCCGAACGUGUCACGUTT-3'; TFEB, 5'-TGTTGGTCATCCAGGCG-3' (Obio, Shanghai, China). Knocking down efficiency was analyzed by western blot analysis and PCR according to our previous studies 5, 11.

### Animal study

*In vivo* study was carried out by a subcutaneous nude mouse model. Briefly, 105 LN229 cells were injected subcutaneously into the right shoulder of 4-week-old nude mice (SLAC laboratory animal Center; Shanghai, China). 2 weeks later, nude mice (n = 10) were divided into two groups, control group and FIN56 treatment group. Subcutaneous tumors were harvested 30 days after treatment. Tumors were fixed and paraffin-embedded for immunohistochemistry.

### Immunohistochemistry

Paraffin-embedded tumor tissues were sectioned (4 μm) and mounted onto microscopic slides. Deparaffinized sections were incubated with the primary antibody at 4 °C overnight (4-HNE, Abcam), rinsed with PBS, and incubated with goat anti-rabbit secondary antibody (Beyotime; Shanghai, China). Images were taken by a Leica DMi8 microscope (Leica Microsystems). Staining of cancer cells was scored as follows: 0, no staining; 1, weak staining in <50% cells; 2, weak staining in ≥50% cells; 3, strong staining in <50% cells; and 4, strong staining in ≥50% cells.

### Statistical analysis

Unpaired t-tests were performed using GraphPad Prism software program (Version 6.07; La Jolla, CA, USA). Results are presented as the mean ± SE. *P*-values < 0.05 were considered statistically significant.

## Results

### FIN56 inhibits cell growth in GBM cells

**Figure 1:**
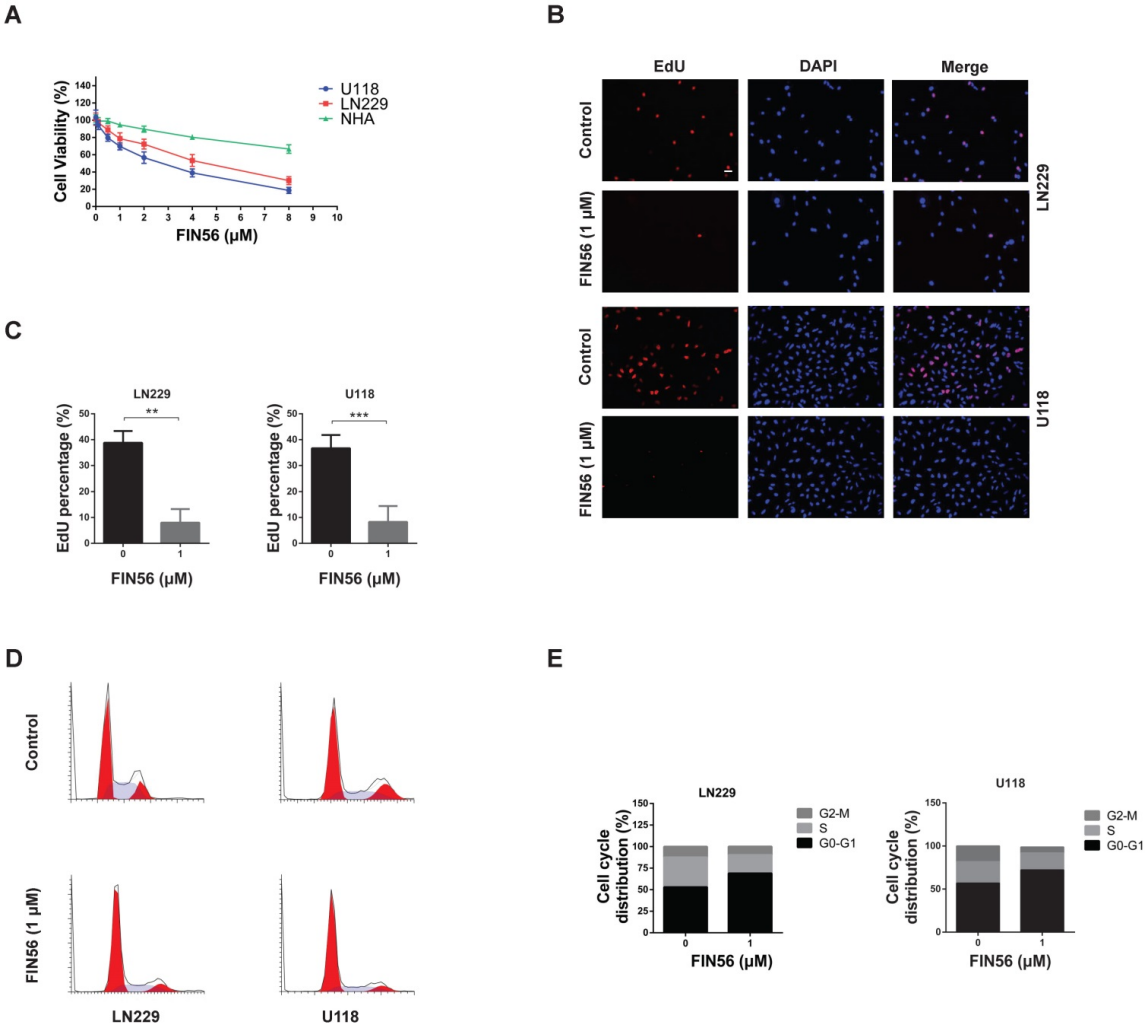
** FIN56 inhibits cell growth of GBM cells (A)** Growth curves generated with colorimetric data (O.D. 450 nm) from the CCK-8 assay. LN229, U118 and normal human astrocytes (NHA) cells were treated with different doses of FIN56 for 24 h. **(B)** Fluorescence images of EdU of 1 μM FIN56 or DMSO (control) for 24 h in LN229 and U118 cells. **(C)** Quantification of EdU positive cells in (B). **(D)** Cell cycle analysis of LN229 and U118 cells treated with 1 μM FIN56 or DMSO (control) for 24 h. **(E)** Quantification of cell cycle parameters G0-G1, S, and G2-M obtained from flow cytometric analysis in D. **P < 0.01; ***P < 0.001; scale bars: 50 µm.

Firstly, we tested the anti-tumor effect of FIN56 on GBM cells *in vitro*. CCK-8 was used to test the cell viability after different doses of FIN56 treatment and results showed that FIN56 decreased the cell viability of LN229 and U118 cells in a dose-dependent manner (Fig. 1A). The IC_50_ for LN229 and U118 were 4.2, 2.6 µM respectively (Fig. 1A). Normal human astrocytes (NHA) were used as normal control and results showed that glioma cells were more sensitive to FIN56 than NHA (Fig. 1A). Then we used EdU assay to test the cell proliferation after FIN56 treatment. We found that FIN56 decreased cell proliferation significantly compared with control group (Fig. 1B and 1C). Cell cycle arrest is one of the most important factors contributing to the inhibition of cell proliferation. We found that after FIN56 treatment, cell cycle of LN229 and U118 was arrested at GO/G1 phases (Fig. 1D and 1E). All these results suggested that FIN56 had promising anti-tumor effect on GBM cells.

### FIN56 induces ferroptosis in GBM cells

**Figure 2:**
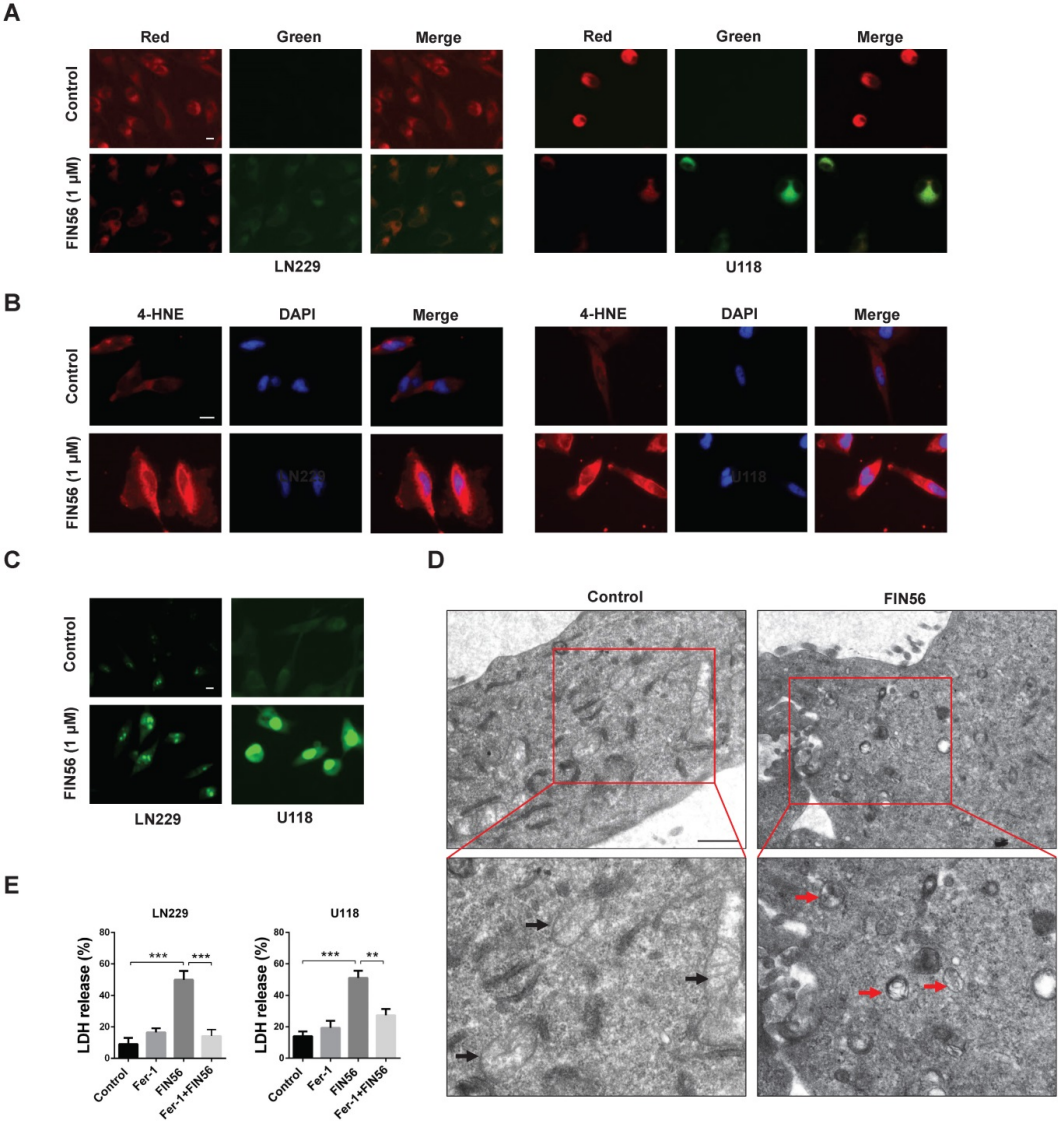
** FIN56 induces ferroptosis in GBM cells. (A)** Images of BODIPY581/591 C11 staining of LN229 and U118 cells treated with 1 μM FIN56 or DMSO (control) for 24 h. **(B)** Images of immunofluorescence staining of 4-HNE of LN229 and U118 cells treated with 1 μM FIN56 or DMSO (control). **(C)** Images of CellRox green staining of LN229 and U118 cells treated with 1 μM FIN56 or DMSO (control). **(D)** Ultrastructure of LN229 cells treated with 1 μM FIN56 or DMSO (control) by transmission electron microscopy. Mitochondria were pointed out in DMSO (black arrows) and FIN56 (red arrows) treatment group. **P < 0.01; ***P < 0.001. Size bars in (A), (B) and (C): 10 µm, in (D): 0.5 µm.

Previous studies recognized FIN56 as a ferroptosis inducer. In our study, we tried to figure out whether FIN56 induced ferroptosis in GBM cells. Lipid peroxidation is one of the most important features of ferroptosis. Lipid peroxidation was assessed by BODIPY^581/591^/C11, a free radical sensor. When the levels of lipid peroxidation increase, red fluorescence of BODIPY^581/591^ /C11 decreases and green fluorescence shows up. FIN56 treatment increased green fluorescence and red fluorescence decayed (Fig. 2A). Immunofluorescence revealed that FIN56 increased the expression of 4 hydroxynonenal (4-HNE), an aldehydic product of lipid peroxidation (Fig. 2B). We also found that FIN56 also increased ROS production compared with control group (Fig. 2C). The ultrastructure of GBM cell lines LN229 and U118 was studied by transmission electron microscope. Fig 2D indicated that FIN56 induced the shrink of the mitochondria and increased the density of its membrane (red arrows). To test whether the cytotoxic effect is ferroptosis-dependent, LDH release assay was carried out and results demonstrated that Ferrostatin-1 (Fer-1), a ferroptosis inhibitor, obviously inhibited FIN56-induced cell death (Fig. 2E). All these results illustrated that FIN56 induced ferroptosis in GBM cells.

### FIN56 causes lysosomal membrane permeabilization in a ferroptosis-dependent manner

Our previous study showed that disulfiram, a novel ferroptosis inducer, induced LMP in GBM cells. Here, we tried to figure out whether FIN56 had the same effect.

**Figure 3:**
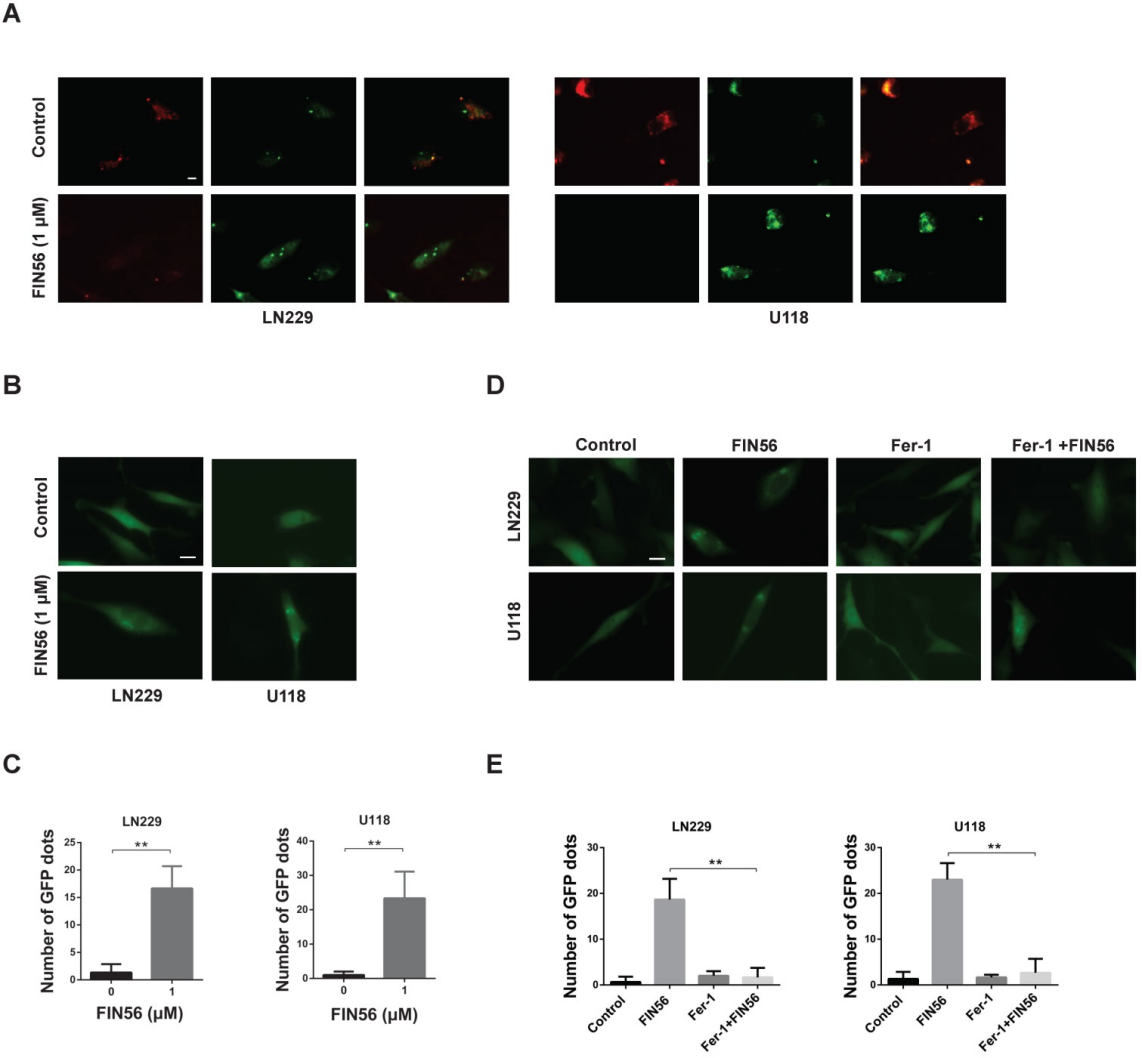
** FIN56 causes lysosomal membrane permeabilization in a ferroptosis-dependent manner. (A)** Fluorescence images of acridine orange staining of LN229 and U118 cells treated with 1 μM FIN56 or DMSO (control). **(B)** Fluorescence images of EGFP-Gal3 transfection of LN229 and U118 cells treated with 1 μM FIN56 or DMSO (control). **(C)** Quantification of green puncta in (B). **(D)** Fluorescence images of EGFP-Gal3 transfection of LN229 and U118 cells treated with DMSO (control), 1 μM FIN56 or, 2 µM ferrostatin-1 (Fer-1) or 2 µM ferrostatin-1 (Fer-1) combined with 1 μM FIN56. **(E)** Quantification of green puncta in (D). **P < 0.01; scale bars: 10 µm.

Acridine orange (AO), a lysosomotropic dye, is enriched in intact lysosomes, which emits red fluorescence. While in impaired lysosomes, the enrichment of AO is interfered, and red fluorescence intensity decreases. Fig. 3A illustrated that FIN56 decreased red fluorescence compared with control group.

When the membranes of lysosomes are impaired, Galectin 3, a β-galactoside binding lectin, accumulates on luminal glycoproteins and is recognized as a better method to monitor the occurrence of LMP. LN229 and U118 cells were transfected with a GFP-fused Galectin 3 (EGFP-Gal3) plasmid and results showed that FIN56 treatment increased green fluorescent dots, compared with control group (Fig. 3B and 3C). In the process of ferroptosis, levels of lipid peroxidation and ROS increase. ROS accumulation is one of the most important factors leading to LMP. Our previous study also showed that ferroptosis inducer disulfiram contributed to LMP in a ferroptosis and ROS dependent manner. Here, ferroptosis was inhibited by Fer-1 and EGFP-Gal3 dots decreased obviously in Fer-1 and FIN56 combined treatment, compared with FIN56 treatment alone (Fig. 3D and 3E). All these data demonstrated that FIN56 triggered LMP in a ferroptosis manner in GBM cells.

### FIN56 induces LMP in a TFEB dependent manner

**Figure 4:**
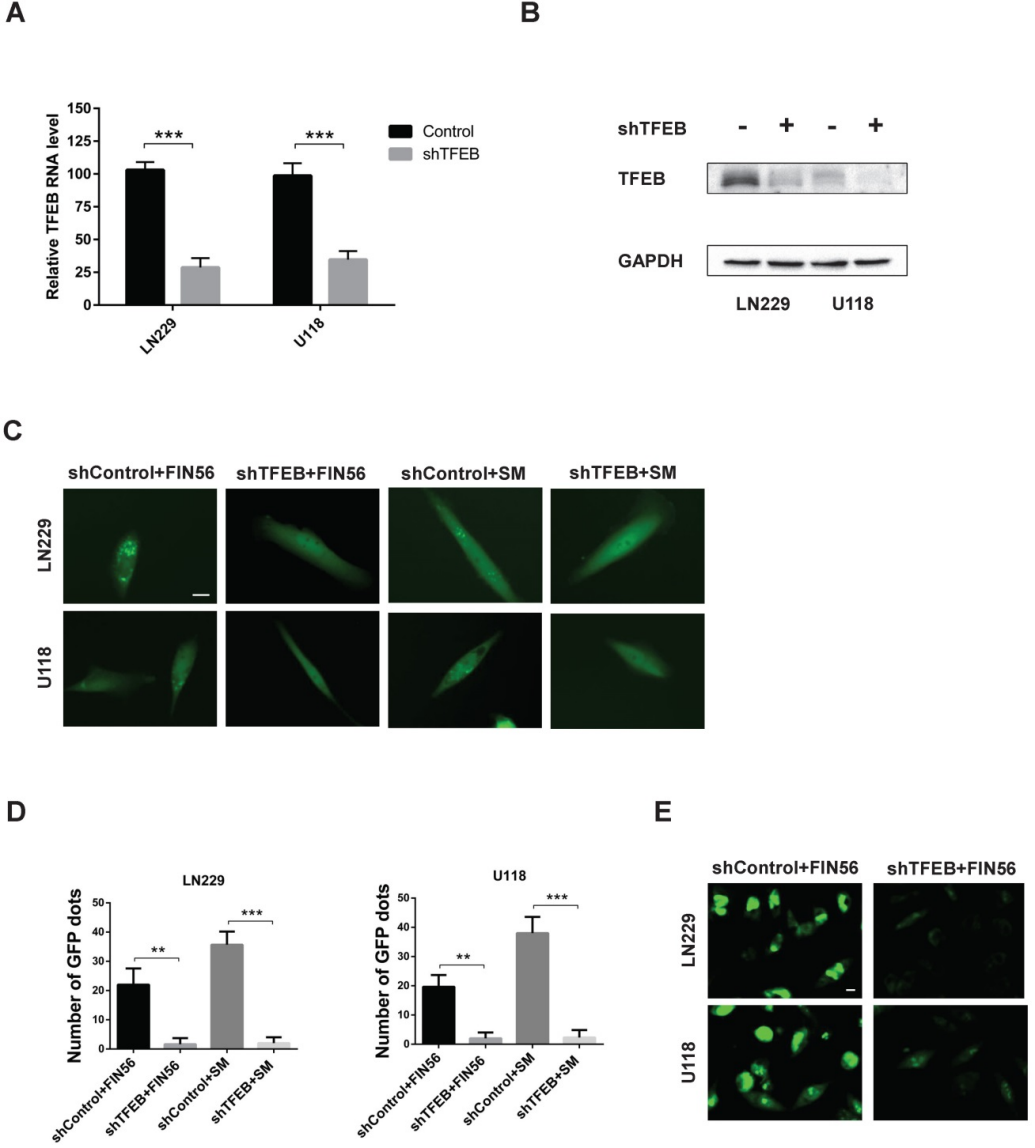
** FIN56 induces LMP in a TFEB dependent manner. (A)** Reverse transcription PCR of LN229 and U118 cells transfected with shControl or shTFEB. **(B)** Western blot analysis of LN229 and U118 cells transfected with shControl or shTFEB. **(C)** Fluorescence images of EGFP-Gal3 transfection of LN229 and U118 cells treated with shControl combined with 1 μM FIN56, shTFEB combined with 1 μM FIN56, shControl combined with 5 μM siramesine (SM), shTFEB combined with 5 μM SM. **(D)** Quantification of green puncta in (C). **(E)** Images of CellRox green staining of LN229 and U118 cells treated with shControl combined with 5 μM siramesine (SM), shTFEB combined with 5 μM SM. **P < 0.01; ***P < 0.001; size bars: 10 µm.

When LMP occurs, hydrolases are released from damaged lysosomal membrane and cell death is induced. There are more than 50 acid hydrolases in the lumen of lysosomes. Among all the hydrolases involved in lysosomal cell death, cathepsin B and cathepsin D are the most important. Our previous study found that knocking down transfactor EB (TFEB), the expression of cathepsin B and cathepsin D was inhibited 11. Besides, TFEB played an important role in the biogenesis of lysosome. As a result, we assumed that TFEB might contribute to LMP. To test our assumption, we knocked down TFEB with a specific siRNA and the knocking-down efficiency was verified by reverse transcription PCR and western blot analysis (Fig. 4A and 4B). Then we added FIN56 in control or TFEB knocking-out LN229 and U118 cells. Fig. 4C and 4D showed that after FIN56/siramesine treatment, EGFP-Gal3 dots decreased significantly in TFEB knocking-out LN229 and U118 cells compared with control group. We also found that after knocking down TFEB, FIN56 didn't increase ROS production in LN229 and U118 cells (Fig. 4E). These data showed that FIN56 induced LMP in a TFEB dependent manner. Further study also illustrated that when cathepsin B and cathepsin D were inhibited by their inhibitor, CA-074 Me, pepstatin A, FIN56 didn't induce LMP in LN229 and U118 cells (supplementary Fig. 1), which suggested that FIN56-induced LMP was cathepsin B- and cathepsin D-dependent.

### FIN56 inhibited cell growth and induced ferroptosis *in vivo*

**Figure 5:**
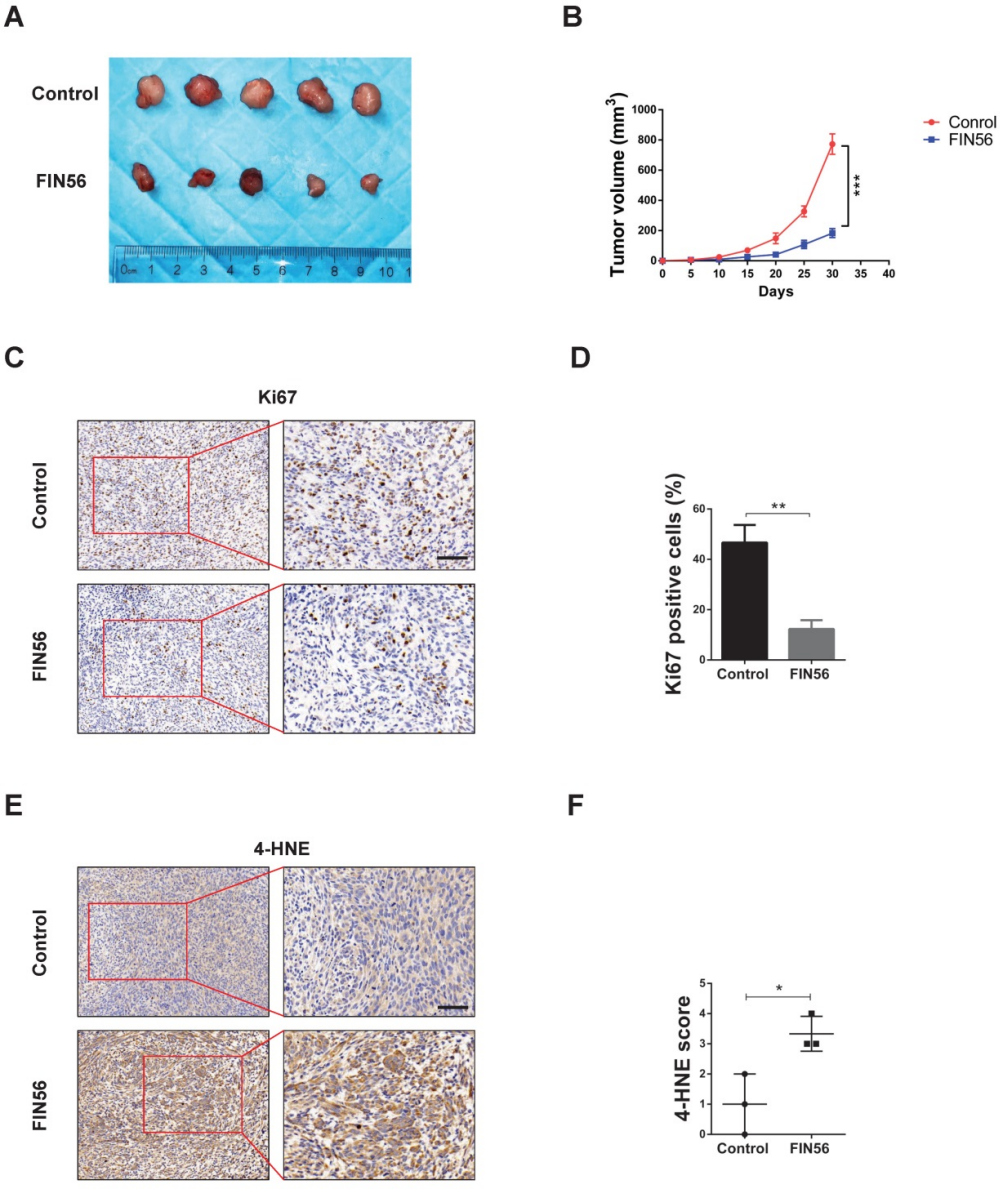
** FIN56 inhibited cell growth and induced ferroptosis *in vivo*. (A)** Images of subcutaneous tumors after control or FIN56 treatment. **(B)** Quantification of tumor volume in (A). **(C)** Immunohistochemistry staining of ki67 to determine cell proliferation. **(D)** Quantification of (C). **(E)** Immunohistochemistry staining of 4-HNE to determine cell ferroptosis. **(F)** Quantification of (C). *P < 0.05; **P < 0.01; ***P < 0.001; size bars: 50 µm.

Finally, we tested the anti-tumor effect of FIN56 *in vivo*. We implanted LN229 cell subcutaneously in nude mice and treated them with or without FIN56. 30 days later tumors were harvested and tested by immunohistochemistry. Results showed that FIN56 decreased tumor volume obviously (Fig. 5A and 5B). Ki67 is a marker to test proliferation ability and immunohistochemistry results demonstrated that FIN56 treatment decreased Ki67 positive cells (Fig. 5C and 5D). FIN56 also significantly increased protein levels of 4-HNE (Fig. 5E and 5F). All those results illustrated that FIN56 had efficient anti-tumor effect *in vivo*.

## Discussion

Although more and more attention has been paid to the studies of glioma, few breakthroughs have been achieved. Apoptosis is considered as the most important signaling pathway of various methods of anti-tumor treatment 4. However, tumor cells will develop apoptotic resistance after repeated treatment, and it is one of the most important factors contributing to the relapse and poor prognosis of glioma 17.

Among many types of programmed cell death, ferroptosis is formally denominated in 2012^18^. Until now, studies on ferroptosis keep increasing. Ferroptosis plays an important role in various types of diseases 19-21. Overactive ferroptosis triggers tissue injury in diseases like cardiovascular/cerebrovascular diseases and neurodegenerative diseases 22. While in cancer, inactivation of ferroptosis contributes to tumor growth and resistance to chemo- and radio-therapy 23. As a result, ferroptosis inducers are promising anti-tumor reagents.

Here, in our study we found that FIN56, a novel ferroptosis inducer, showed efficient anti-glioma effect both *in vitro* and *in vivo*. Firstly, FIN56 decreased cell viability in GBM cell lines LN229 and U118. FIN56 also inhibited cell proliferation by causing cell cycle arrest. As a novel ferroptosis inducer, FIN56 induced cell death in LN229 and U118 cells in a ferroptosis-dependent manner. We also found that FIN56 triggered LMP in LN229 and U118 cells. Further studies illustrated that the LMP induced by FIN56 was ROS and TFEB dependent. At last, *in vivo* studies showed that FIN56 had efficient anti-tumor effect in a nude mice model. But whether FIN56 will penetrate the blood brain barrier is still unknown. More studies are warranted to test the anti-tumor effect of FIN56 on orthotopic glioma xenografts.

Lysosomal membrane permeabilization (LMP) is a lysosome-dependent cell death. A study by Gao et al showed that ferroptosis is a lysosomal cell death process 13. In our previous study, we also found that disulfiram, traditionally used for the treatment of alcoholism, induced ferroptosis and LMP in glioma cell lines 5. As a result, ferroptosis is closely related to LMP, and lysosomes might be the key link. Previously, lysosome is recognized as the waste bag for its role in late stage of autophagy 9. But more and more studies demonstrate that lysosome plays an important role in regulating intracellular signaling pathways 24, 25. And TFEB, a family of basic helix-loop-helix leucine zipper transcription factors, is recognized as one important regulator of lysosomal biogenesis 26-28. TFEB has also been reported to be involved in clearance of oxidative stress 29. A study by Park et al showed that TFEB activates Nrf2, an important factor in regulating oxidative stress, by increasing its stability 30. Considering the role of TFEB in lysosomal biogenesis and oxidative stress in the initiation of LMP, we assumed that TFEB might be involved in LMP. In our study, we found that FIN56 induced LMP, and this process was inhibited by knocking down TFEB. Siramesine-induced LMP could also be inhibited by knocking down TFEB. But whether TFEB regulates ferroptosis is not known. Further studies are warranted in the future.

In summary, our study identified that FIN56, a novel ferroptosis inducer, showed significant anti-tumor effect and triggered lysosomal membrane permeabilization in a TFEB-dependent manner in glioma. FIN56 is a promising anti-tumor compound. More studies are needed on other tumor types.

## Supplementary Material

Supplementary figure.
