# Supplementary material for: FIN56, a novel ferroptosis inducer, triggers lysosomal membrane permeabilization in a TFEB-dependent manner in glioblastoma
Source: J Cancer. 2021 Sep 13;12(22):6610–9. doi: 10.7150/jca.58500 (PMC8517990; doi:10.7150/jca.58500)

Supplementary Figure 1. When cathepsin B and cathepsin D were inhibited by their inhibitor, CA-074 Me, pepstatin A, FIN56 didn't induce LMP in LN229 and U118 cells. Size bars = 10 μm.

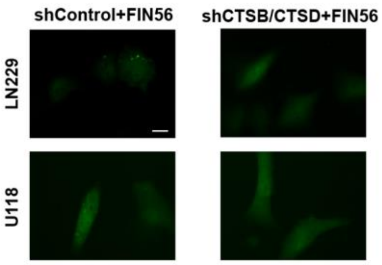

Supplement: Supplementary file 1 — Supplementary figure. [file jcav12p6610s1.pdf]
